# On a Variety of False Aneurism

**Published:** 1843-03-04

**Authors:** 


*On a Variety of False Aneurism.* By Robert Liston, F. R. S., Surgeon to University College Hospital, Profesor of Clinical Surgery, &c., &c. (Read to the Royal Medical Chirurgical Society, March 8th, 1842. Printed at the author's expense, for distribution among the members.) London: 8vo. pp. 39.

This valuable and instructive paper was refused by the Controlling Council of the Royal Medical Chirurgical Society, and its author, in justice to himself, has published it at his own expense.

In a delicate boy, about nine years of age, after an attack of cough and fever, a small swelling formed below the right ear, which was fomented and poulticed, about two months before his admission into the hospital. A few days before his admission, there was a rapid increase in size, and its shape became irregular. When he entered the hospital, there was a tumour at the angle of the jaw of the right side. Backwards it extended nearly to the posterior border of the sterno-cleido-mastoid muscle—where it seemed to point—the upper part of which was pushed forwards; downwards, to within an inch of the clavicle; and forwards, to about half the length of the horizontal ramus of the lower jaw. Both respiration and deglutition were impeded by its projection into the mouth between the arches of the palate. There was indistinct fluctuation, and slight pulsation was detected immediately over the carotid artery; but on grasping the sides of the tumour, no pulsation could be discovered, nor could any be felt inside the mouth. Looking upon it as an abscess, Mr. Liston opened it with a bistoury; a gush of blood followed, of which about four ounces was lost in a few seconds. The wound was immediately closed by hare-lip pins and the twisted suture, and the hæmorrhage controlled. Next day the common carotid was tied, after a deep and difficult dissection, immediately above the innominata. The swelling decreased, and all appeared to do well until the thirteenth day, when secondary hæmorrhage occurred, and the boy died on the fifteenth day. On dissection, the tumour was found to contain a quantity of dark, grumous blood, external to which was a thin layer of organized lymph, which entirely lined the parietes of the cyst.

"A probe was passed down the internal carotid artery, and found to enter the cyst just opposite the division of the common carotid; this point was, however, obstructed, a mass of coagulable lymph almost entirely blocking up the entrance. The parietes of the tumour behind, were about a line or more in thickness, but on the outer side, on the aspect adjacent to the puncture, they were much thinner. They appeared to be composed of three layers differing in character, but organically connected together. The outermost layer consisted of condensed cellular tissue having portions of the surronnding structures attached to or imbedded in it. The middle layer, which was not so evident on the outer side of the tumour, was very dense and opaque, so that it appeared like a distinct white line on a section of the parietes of the tumour. The innermost layer was soft and pulpy, semi-transparent, and of a pale dirty red colour; its inner aspect, forming the inner surface of the cyst, was smooth, and for the most part even; but in some points, and especially at the posterior part of the cyst, it presented a fasciculated appearance like the interior of the auricles of the heart, or of a fasciculated bladder, only not so well marked; it consisted, as I have said, of a flaky lymph-like substance throughout. Opposite the outer part of the tumour (where its parietes were thinnest) this substance was flocculent and broken, and contained patches of a bright yellow colour; here it was not distinctly laminated, and it adhered rather firmly to the middle layer of the cyst, but in other situations it was easily separable into laminæ, and was not so firmly adherent. The cavity of the cyst contained a quantity of grumous blood. The common carotid and the internal and external carotid were firmly attached to the front of the tumour. The opening by which the cyst had communicated with the artery had been about three lines wide and two and a half lines long, and was situated at the bifurcation of the common carotid. It was now completely closed by a firm clot, in which no perforation was visible, so that the probe which had been passed down the internal carotid into the cavity, must have been forced either on one side of this clot or through it. On the side next the vessel, the surface of the clot was concave, broken in the centre, but smooth towards the circumference, where it was closely adherent to the margin of the opening in the carotid artery. The opposite side of the clot was convex, and projected into the cavity of the cyst. On making a vertical section through the artery, the walls of the tumour, and the clot, the latter was seen to be composed of fibrinous laminæ. The edges of the opening in the vessel were found to be well defined and slightly everted; the external coat of the artery was distinctly traced, and afterwards dissected from the middle coat quite up to the margin of the opening, where it terminated abruptly, not being reflected on to the outer surface of the tumour. The coats of the vessel showed not the slightest dilatation at the part where it was connected with the tumour."

From the dissection, Mr. Liston thinks he has succeeded in establishing the fact that his original diagnosis was correct, and his proceeding justifiable. There can be little doubt, he believes, that the tumour was originally a scrofulous abscess developed in the lymphatic cervical ganglia, close to the great vessels, which it compressed; that finally ulceration of the arterial coat occurred, and that the cyst of a chronic abscess was thus converted into the sac of a false aneurism. This view gains additional strength from the fact of the extreme rarity of true aneurism in children—from the progress of the disease—its rapid development, and maturity, so to speak, in two months—its accumulation behind the posterior border of the sterno-mastoid muscle, the absence of disease or dilatation in the artery, and, finally, the appearance of the opening in the vessel—ragged, abrupt, well defined, and having all the appearance of recent ulceration.

Why the operation was delayed until the day following the accident, we cannot conceive. Good surgery we think would have demanded its immediate performance, and the deep and difficult dissection subsequently encountered, due doubtless, in a great measure, to infiltration, would have been avoided.

Mr. Liston has appended eight analogous cases. The first was one of Dr. Craigie's, where an extensive abscess of the tonsil opened into the sheath of the carotid vessels, and finally communicated with the common carotid, close to its bifurcation. In another case the femoral artery was opened. In a third, the common carotid was penetrated by a pharyngeal abscess.

The subject is highly important as a question of diagnosis, and will, it is hoped, receive additional elucidation by the same course of other surgeons, in the publication of similar cases.

